# Supplementary material for: Structure and activation mechanism of the Makes caterpillars floppy 1 toxin
Source: Nat Commun. 2023 Dec 12;14:8226. doi: 10.1038/s41467-023-44069-2 (PMC10716152; doi:10.1038/s41467-023-44069-2)
Supplement: Supplementary file 1 — Supplementary Information [file 41467_2023_44069_MOESM1_ESM.pdf]

Structure and activation mechanism of the Makes caterpillars floppy 1 toxin

Alexander Belyy[1,*], Philipp Heilen[1,*], Philine Hagel[1], Oliver Hofnagel[1], and Stefan Raunser[1,#]

[1]Department of Structural Biochemistry, Max Planck Institute of Molecular Physiology, Otto-Hahn-Str. 11, 44227 Dortmund, Germany

*The authors contributed equally.

#Correspondence should be addressed to: stefan.raunser@mpi-dortmund.mpg.de.

Supplementary information

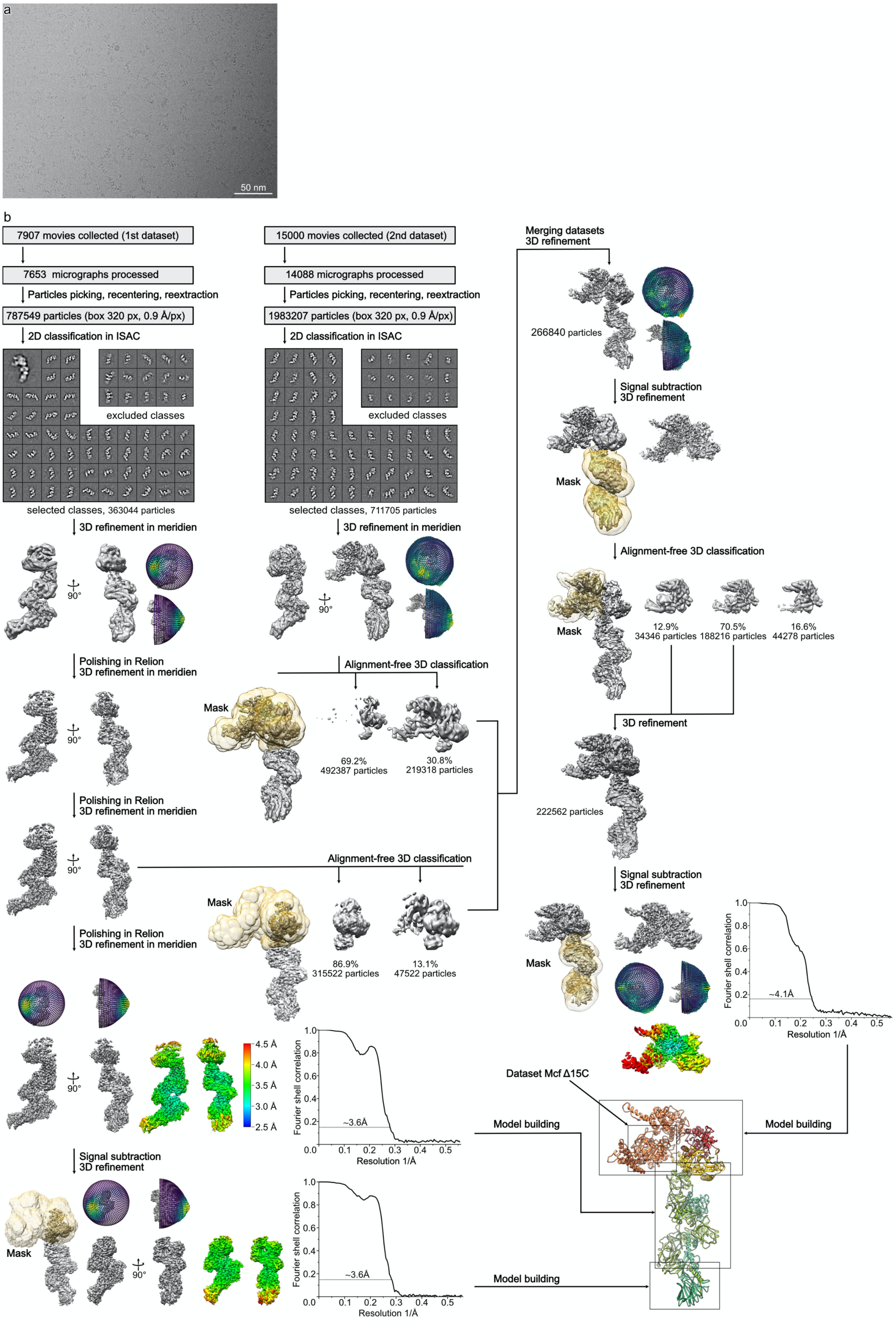

Supplementary Fig. 1. Processing of the full-length Mcf1 dataset. **a** An example of analyzed cryo-EM micrographs. **b** Processing overview with angular distributions and Fourier shell correlation curves of the final reconstructions used for model building.

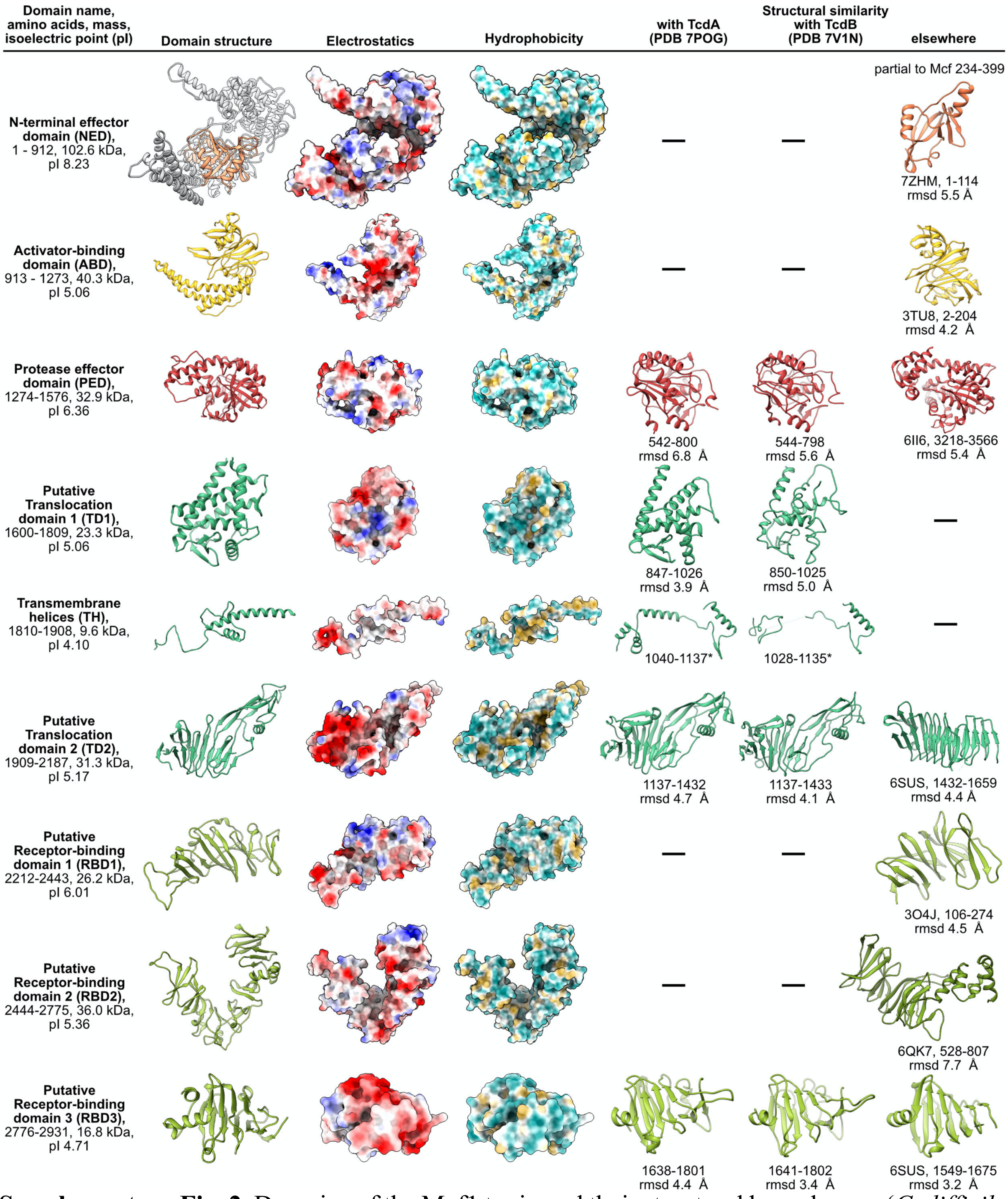

Supplementary Fig. 2. Domains of the Mcf1 toxin and their structural homologues (*C. difficile* TcdA toxin – PDB 7POG[1]; *C. difficile* TcdB toxin – PDB 7V1N[2]; *P. aeruginosa* RhsP2 toxin – PDB 7RT7[3]; *Burkholderia* lethal factor – PDB 3TU8[4]; *Vibrio vulnificus* Mcf-like domain – PDB 6II6[5]; RTX domains of *Bordetella pertussis* CyaA toxin – PDB 6SUS[6]; Acylaminoacyl peptidase – PDB 3O4J[7]; Elongation protein complex 1 – PDB 6QK7[8]) with rmsd calculated by *cealign* in PyMOL (v2.5.2[9]).

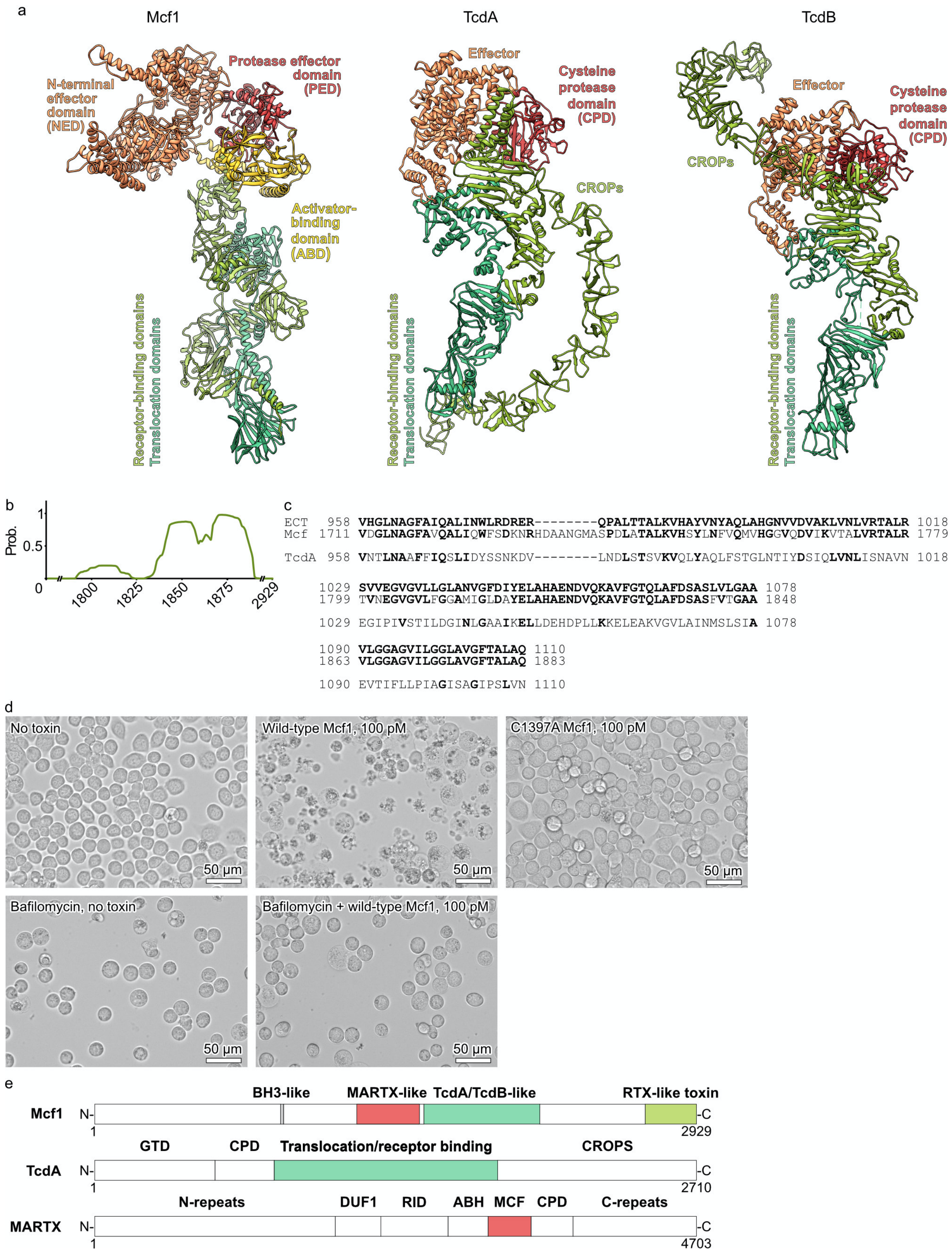

Supplementary Fig. 3. Mcf1 is a member of the superfamily of large clostridial toxins. **a** Structural comparison of Mcf1 with TcdA (PDB 7POG[1]) and TcdB (PDB 7V1N[2]). **b** Computational analysis of potential transmembrane regions in Mcf1 by TMHMM 2.0 [10]. Prob. – amino acid probability score of being part of a transmembrane helix. Source data are provided as a Source Data file. **c** Consensus sequence alignment of evolutionary conserved translocase (ECT) of large clostridial toxins, Mcf1 and TcdA. **d** Toxicity of Mcf1 in insect Sf9 cells. The experiment was performed twice. **e** Schematic representation of homologous regions in Mcf1,

TcdA from *C. difficile* and MARTX from *V. vulnificus* strain MO6-24/O[5]. GTD – glycosyltransferase domain, CPD – cysteine protease domain, CROPS – combined repetitive oligopeptides, DUF1 – domain of unknown function, RID – Rho inactivation domain, ABH – α/β-hydrolase domain, MCF – Makes caterpillars floppy-like domain.

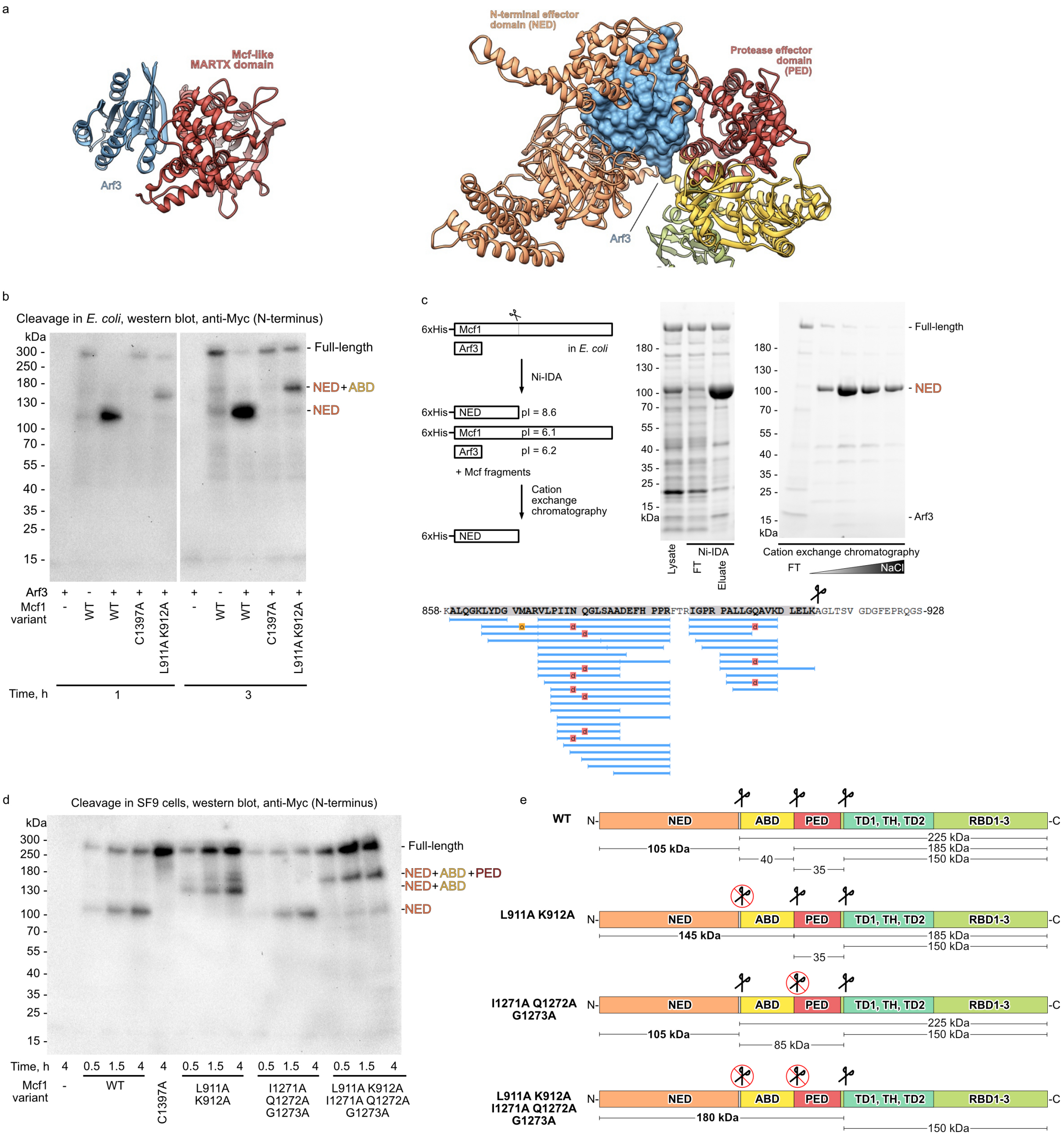

Supplementary Fig. 4. Autoprocessing of Mcf1. **a** Arf3 cannot bind to the protease effector domain of Mcf1 in the same way that it binds to the Mcf-like MARTX domain (PDB 6II6, [5]) due to the sterical clash with the N-terminal effector domain. **b** Western blot (WB) analysis of lysates of *E. coli* coexpressing Arf3 and Myc-tagged Mcf1 variants. **c** Purification of the N-terminal effector domain (NED) after coexpression of Arf3 and the full-length Mcf1 in *E. coli* (above) and the peptide coverage of the C-terminal region of the effector in liquid chromatography with tandem mass spectrometry (below). Source data are provided as a Source Data file. **d** Western blot analysis of N-terminally Myc-tagged Mcf1 autoproteolysis during cell intoxication. All SDS-PAGE and western blot analyses were performed twice. **e** Schematic illustration of the Mcf1 cleavage pattern. IDA – Iminodiacetic acid. Abbreviations: see Fig. 1.

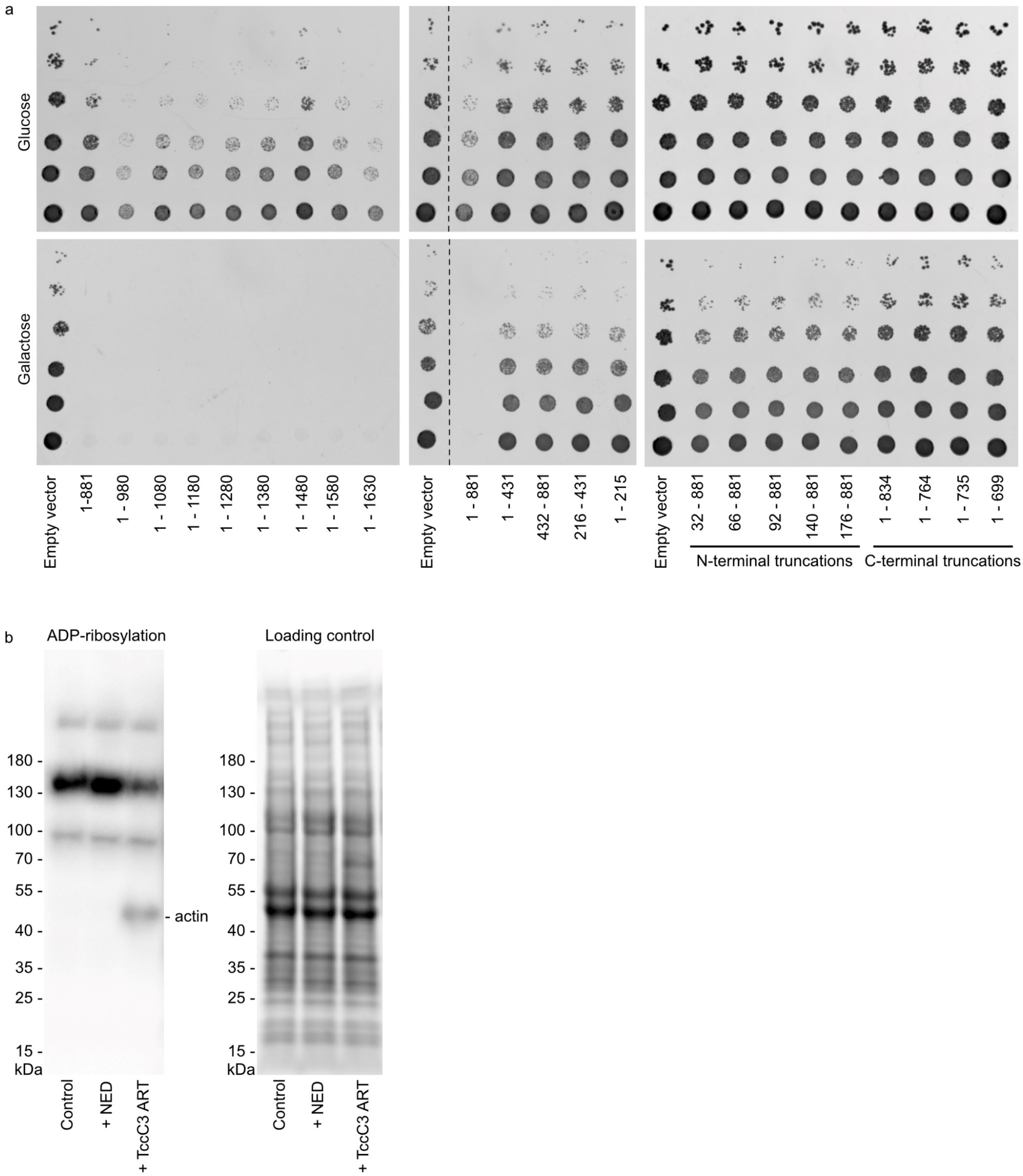

Supplementary Fig. 5. Toxicity of Mcf1 fragments in *S. cerevisiae*. **a** Yeast viability upon expression of N- and C-terminally truncated Mcf1 fragments. In yeast drop tests, *mcf1* fragment expression is stimulated by galactose, and repressed by glucose. **b** ADP-ribosylation analysis of insect cell lysate with the N-terminal effector domain (NED) of Mcf1 and TccC3 ADP-ribosyltransferase (ART) with anti-ADP-ribose binding protein. The reaction and the corresponding western blot were repeated twice.

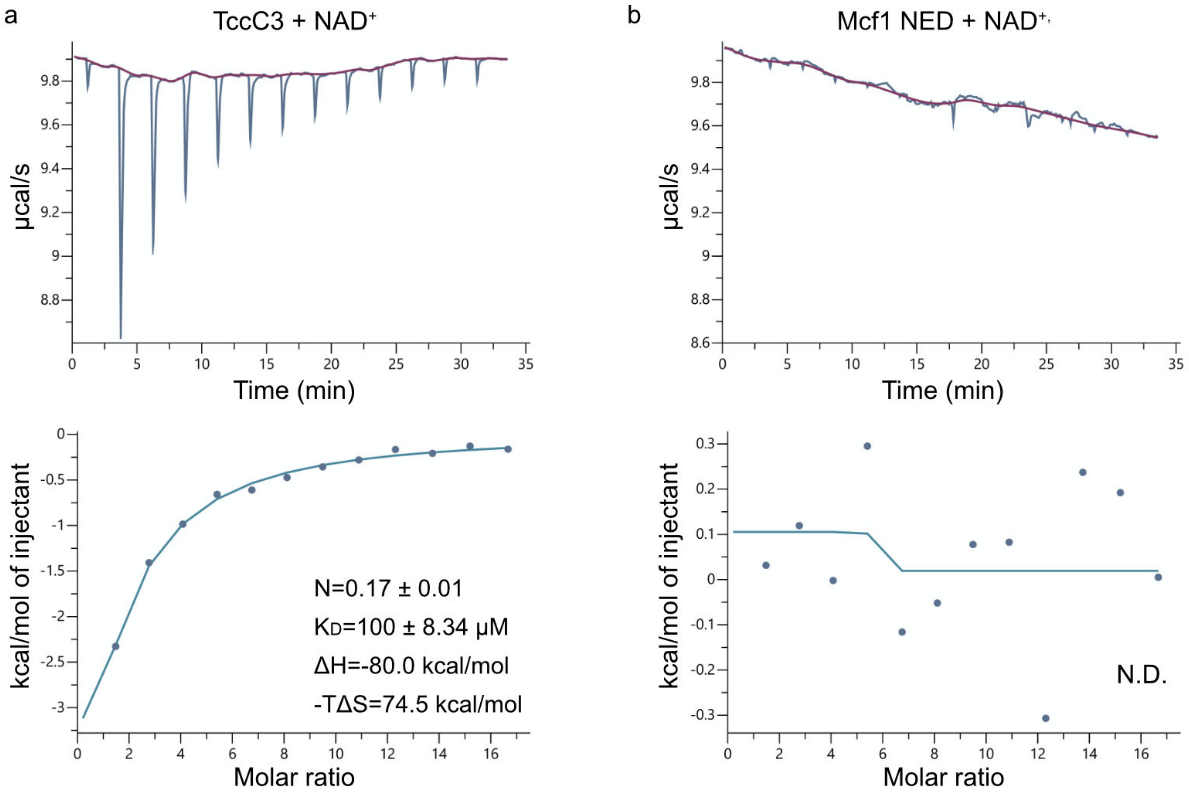

Supplementary Fig. 6. Binding affinity of Mcf1 and NAD^+. ITC measurements of binding affinities by titrating **a** TccC3 (25 µM) or **b** the N-terminal effector domain (NED) (aa 1-914, 25 µM) with NAD^+ (2.2 mM). The integrated heat peaks were fitted with a one-site-binding model to give the binding stoichiometry (N), the enthalpy (ΔH), the entropy (ΔS), and the dissociation constant (K_D). Source data are provided as a Source Data file. The ITC measurements were performed twice.

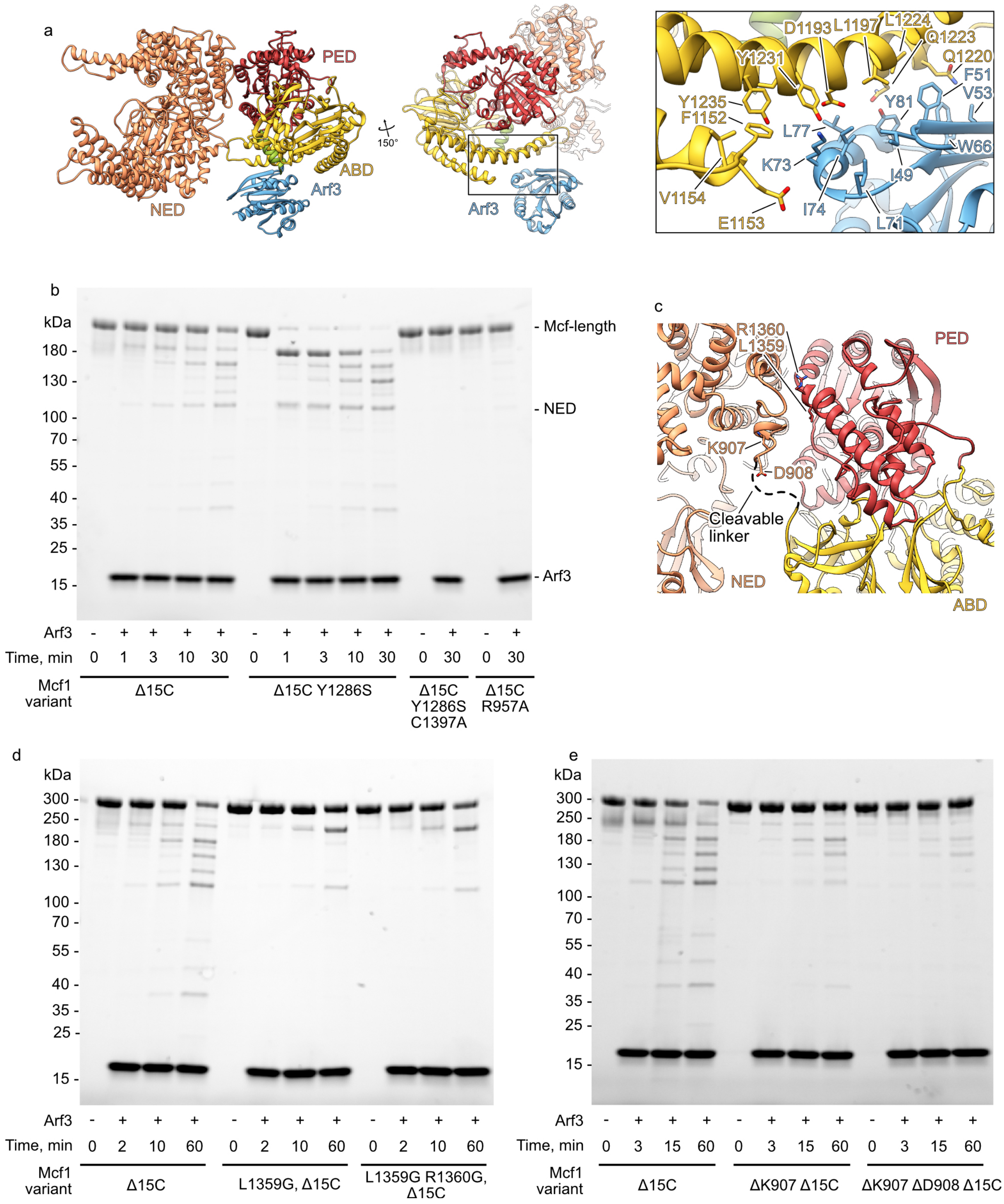

Supplementary Fig. 7. Mechanism of Mcf1 activation. **a** Structure of the Mcf1$_{C1397A\ \Delta 15C}$-Arf3 complex with a focus on the interaction interface. **b, d, e** Autoproteolysis of Mcf1 variants in the presence of Arf3. **c** Position of the cleavable linker-preceding amino acids (K907, D908) and amino acids that create a rigid scaffold of the system (L1359, R1360). Abbreviations: see Fig. 1.

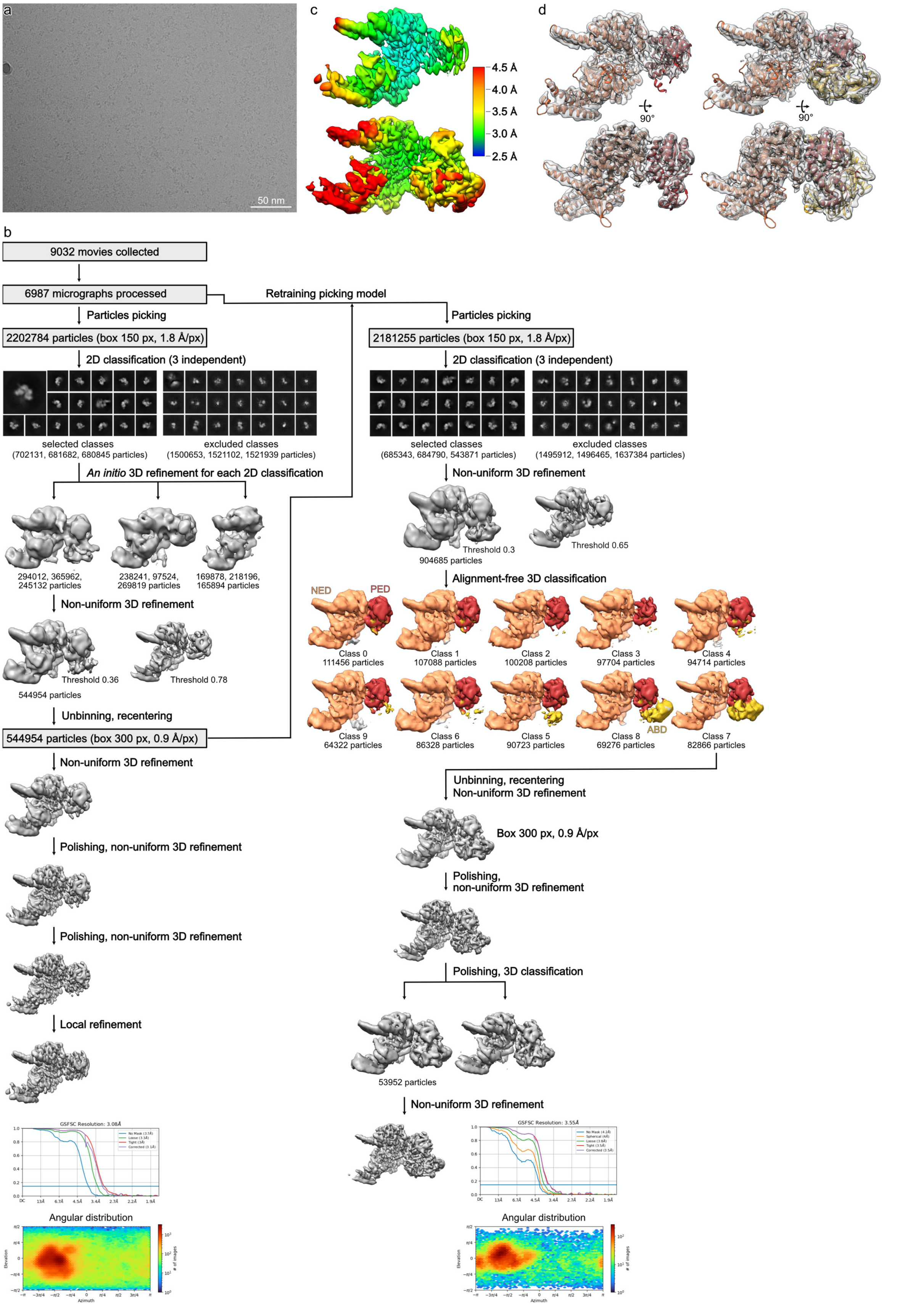

Supplementary Fig. 8. Processing of the Mcf1$_{\mathrm{C1397A\ \Delta 15C}}$ dataset. **a** An example of analyzed cryo-EM micrographs. **b** Processing overview with angular distributions and Fourier shell correlation curves of the final reconstructions used for model building. **c** Local resolution gradient of the final reconstructions. **d** Fit of the molecular models into the cryo-EM densities.

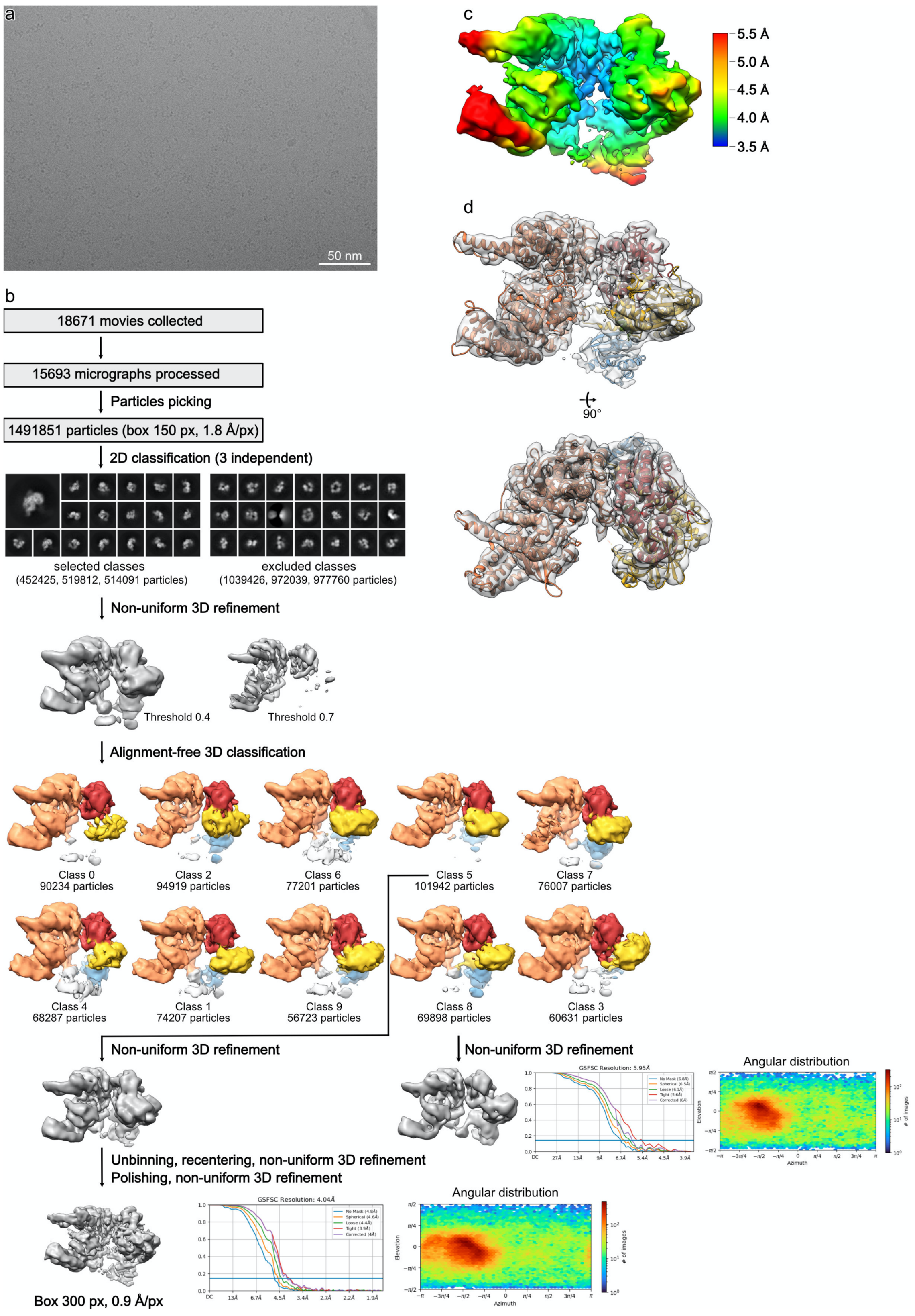

Supplementary Fig. 9. Processing of the Mcf1$_{\mathrm{C1397A\ \Delta15C}}$-Arf3 complex dataset. **a** An example of analyzed cryo-EM micrographs. **b** Processing overview with angular distribution and Fourier shell correlation curves of the final reconstruction used for model building. **c** Local resolution gradient of the final reconstruction. **d** Fit of the molecular model into the cryo-EM density.

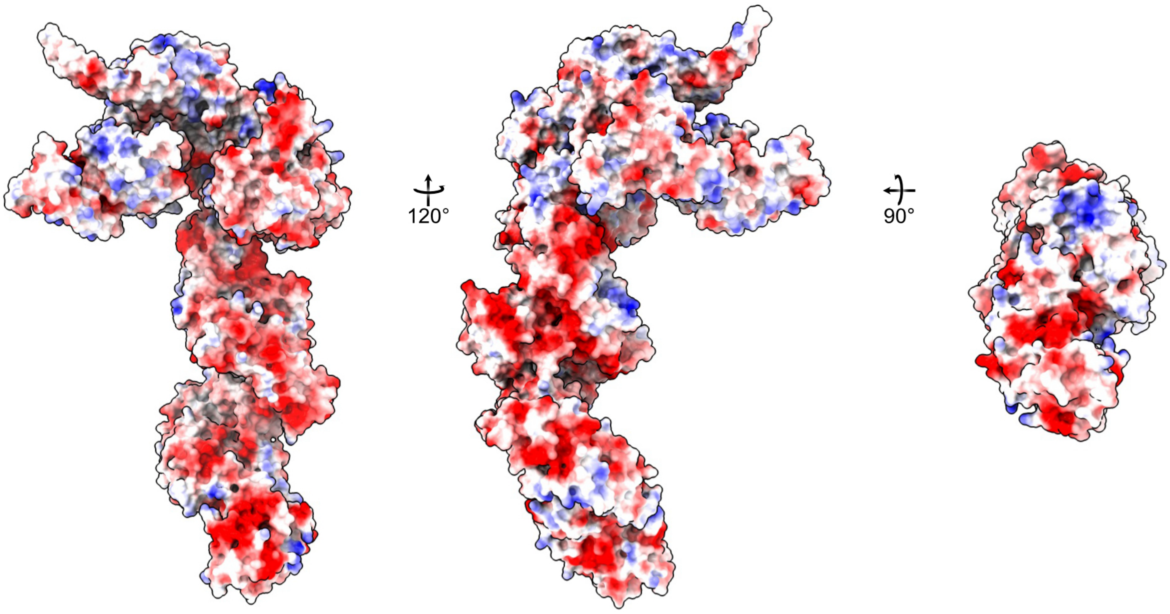

Supplementary Fig. 10. Electrostatic Coulomb potential of Mcf1. Surface of Mcf1 coloured by electrostatic Coulomb potential from −10 kcal mol^{-1} (red) to +10 kcal mol^{-1} (blue).

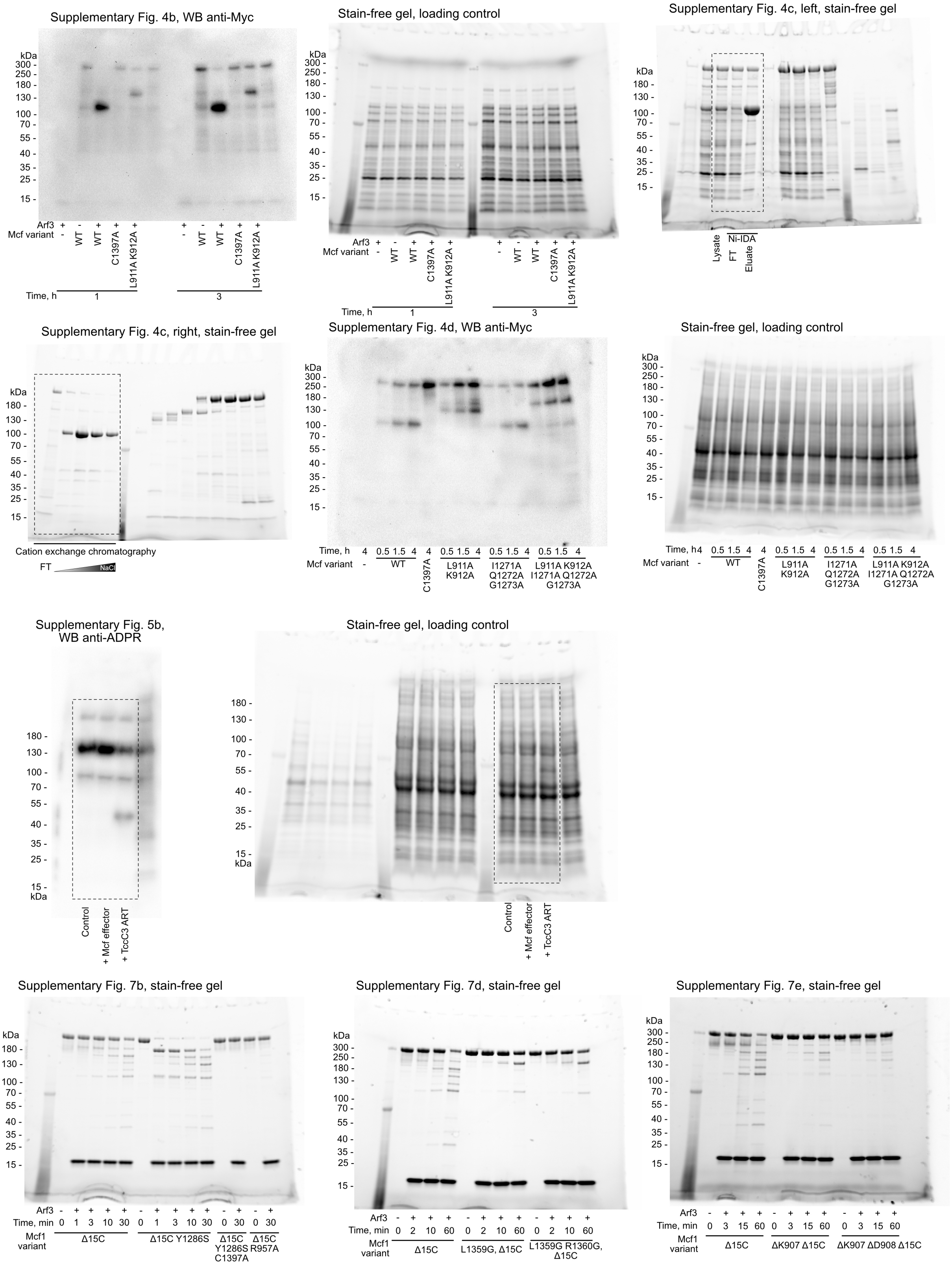

Supplementary Fig. 11. Uncropped SDS-PAGE, western blots (WB) and their loading controls.

Supplementary Table 1. Cryo-EM data collection, refinement and validation statistics

Sample	Full-length Mcf1	Mcf1$_{C1397A\ \Delta15C}$	Mcf1$_{C1397A\ \Delta15C}$-Arf3 complex
Microscope	Titan Krios		
Voltage (kV)	300		
Defocus range (µm)	-1 to -2.5		
Camera	Gatan K3 (super resolution mode)		
Pixel size (Å)	0.45 super resolution mode; 0.9 physical pixel size		
Total electron dose (e/Å^2)	71 and 69	60.85	62.6
Exposure time, s	3	3.5	2
Frames per movie	60	60	60
Number of movies	21,741	9,032	18,671
**3D Refinement**			
Number of particles	363,044 for building the tail region; 219,318 for building the head domain	53,952	101,942
Final resolution (Å)	3.6	3.5	4.0
**Atomic model statistics**			
Non-hydrogen atoms	22,783	12,619	13,714
Molprobity score	1.52	1.51	1.68
Clashscore	3.44	4.21	6.41
Cβ deviations >0.25Å	0	0	0
Bad bonds	0	0	0
Bad angles	0	0	0
Poor rotamers (%)	0	0	0
Favored rotamers (%)	99.71	99.47	99.66
Ramachandran favored (%)	94.40	95.61	95.32
Ramachandran allowed (%)	5.57	4.39	4.68
Ramachandran outliers (%)	0.03	0	0

Supplementary Table 2. **List of primers, strains and plasmids used in this study.**

Bacterial and yeast strains	Description	Reference
E. coli DH5α	F^- Φ80*lac*ZΔM15 Δ(*lac*ZYA-*arg*F) U169 *rec*A1 *end*A1 *hsd*R17(r_k^-, m_k^+) *pho*A *sup*E44 *thi*-1 *gyr*A96 *rel*A1 λ^-	Invitrogen
E. coli BL21 (DE3) CodonPlus RIPL	F^- *ompT hsdS*(r_B^- m_B^-) *dcm*$^+$ Tet^r *gal*λ(DE3) *endA* I [*argU proL* Cam^r] [*argU ileY leuW* Strep/Spec^r]	Agilent
S. cerevisiae MH272-3fα	"Wild-type" strain, *ura3, leu2, his3, trp1, ade2*	[11]
S. cerevisiae Y388	*S. cerevisiae* MH272-3fα + Iota-A [Ade] (p1323)	[12]
S. cerevisiae Y395	*S. cerevisiae* MH272-3fα + empty vector [Ade] (2473)	[13]
S. cerevisiae Y401	*S. cerevisiae* MH272-3fα + Mcf1 fragment 6 (amino acids 1133-1619) [Ade] (pB380)	This study
S. cerevisiae Y438	*S. cerevisiae* MH272-3fα + Mcf1 fragment 6 D1505A [Ade] (pB408)	This study
S. cerevisiae Y439	*S. cerevisiae* MH272-3fα + Mcf1 fragment 6 C1397A [Ade] (pB409)	This study
S. cerevisiae Y452	*S. cerevisiae* MH272-3fα + Mcf1 fragment 6 H1486A [Ade] (pB419)	This study
S. cerevisiae Y639	*S. cerevisiae* MH272-3fα + TcHVR ΔN100 (ART) [Ade] (pB631)	[14]
S. cerevisiae Y772	*S. cerevisiae* MH272-3fα + Mcf1 fragment (amino acids 1-881) [Ade] (pB798)	This study
S. cerevisiae Y773	*S. cerevisiae* MH272-3fα + Mcf1 fragment (amino acids 1-431) [Ade] (pB799)	This study
S. cerevisiae Y774	*S. cerevisiae* MH272-3fα + Mcf1 fragment (amino acids 432-881) [Ade] (pB800)	This study
S. cerevisiae Y775	*S. cerevisiae* MH272-3fα + Mcf1 fragment (amino acids 216-431) [Ade] (pB801)	This study
S. cerevisiae Y776	*S. cerevisiae* MH272-3fα + Mcf1 fragment (amino acids 1-215) [Ade] (pB802)	This study
S. cerevisiae Y788	*S. cerevisiae* MH272-3fα + Mcf1 fragment (amino acids 1-980) [Ade] (pB827)	This study
S. cerevisiae Y789	*S. cerevisiae* MH272-3fα + Mcf1 fragment (amino acids 1-1080) [Ade] (pB828)	This study
S. cerevisiae Y790	*S. cerevisiae* MH272-3fα + Mcf1 fragment (amino acids 1-1180) [Ade] (pB829)	This study
S. cerevisiae Y791	*S. cerevisiae* MH272-3fα + Mcf1 fragment (amino acids 1-1280) [Ade] (pB830)	This study
S. cerevisiae Y792	*S. cerevisiae* MH272-3fα + Mcf1 fragment (amino acids 1-1380) [Ade] (pB831)	This study
S. cerevisiae Y793	*S. cerevisiae* MH272-3fα + Mcf1 fragment (amino acids 1-1480) [Ade] (pB832)	This study
S. cerevisiae Y794	*S. cerevisiae* MH272-3fα + Mcf1 fragment (amino acids 1-1580) [Ade] (pB833)	This study
S. cerevisiae Y795	*S. cerevisiae* MH272-3fα + Mcf1 fragment (amino acids 1-1630) [Ade] (pB834)	This study
S. cerevisiae Y836	*S. cerevisiae* MH272-3fα + *Burkholderia* lethal factor 1 [Ade] (pB990)	This study
S. cerevisiae H009	*S. cerevisiae* MH272-3fα + Mcf1 effector (amino acids 32-881) [Ade] (pH009)	This study
S. cerevisiae H010	*S. cerevisiae* MH272-3fα + Mcf1 effector (amino acids 66-881) [Ade] (pH010)	This study
S. cerevisiae H011	*S. cerevisiae* MH272-3fα + Mcf1 effector (amino acids 92-881) [Ade] (pH011)	This study
S. cerevisiae H012	*S. cerevisiae* MH272-3fα + Mcf1 effector (amino acids 140-881) [Ade] (pH012)	This study
S. cerevisiae H013	*S. cerevisiae* MH272-3fα + Mcf1 effector (amino acids 176-881) [Ade] (pH013)	This study
S. cerevisiae H014	*S. cerevisiae* MH272-3fα + Mcf1 effector (amino acids 1-834) [Ade] (pH014)	This study
S. cerevisiae H016	*S. cerevisiae* MH272-3fα + Mcf1 effector (amino acids 1-764) [Ade] (pH016)	This study
S. cerevisiae H017	*S. cerevisiae* MH272-3fα + Mcf1 effector (amino acids 1-735) [Ade] (pH017)	This study
S. cerevisiae H018	*S. cerevisiae* MH272-3fα + Mcf1 effector (amino acids 1-699) [Ade] (pH018)	This study
S. cerevisiae H023	*S. cerevisiae* MH272-3fα + ABD (amino acids 913-1125) [Ade] (pH023)	This study
S. cerevisiae H024	*S. cerevisiae* MH272-3fα + ABD G1031C (amino acids 913-1125) [Ade] (pH024)	This study

**Plasmids for experiments in *S. cerevisiae***		
2473 YEpGal555	*E. coli*/*S. cerevisiae* shuttle vector [ADE2] with Gal1 promoter	[13]
pB380 YEpGal555 Mcf1 fragment 6	Fragment of the *mcf1* gene, encoding amino acids 1133-1619, was PCR amplified using oligonucleotides TATACTCGAGCAATTCGTCGCCTACCGCGAGC and TATATAGCTAGCGAACCGTGCCCCCCATTGCTCG from pB394, digested with XhoI and NheI and ligated in the digested 2473 YEpGal555 vector.	This study
pB408 YEpGal555 Mcf1 fragment 6 D1505A	The mutation D1505A was introduced by two-step overlap PCR using oligonucleotides TATACTCGAGCAATTCGTCGCCTACCGCGAGC, TATATAGCTAGCGAACCGTGCCCCCCATTGCTCG, CTACTTTTATGCCCCGAACGTCG, and CGACGTTCGGGGCATAAAAGTAG and pB380 as the template. The PCR product was digested with XhoI and NheI, and ligated into 2473 YEpGal555 vector.	This study
pB409 YEpGal555 Mcf1 fragment 6 C1397A	The mutation C1397A was introduced by two-step overlap PCR using oligonucleotides TATACTCGAGCAATTCGTCGCCTACCGCGAGC, TATATAGCTAGCGAACCGTGCCCCCCATTGCTCG, GTGACCGATCTGGTGGCCGTGCTTATCCTTTGGTCAGGGCC, and CTGACCAAAGGATAAGCACGGCCACCAGATCGGTCACCGAC and pB380 as the template. The PCR product was digested with XhoI and NheI, and ligated into 2473 YEpGal555 vector.	This study
pB419 YEpGal555 Mcf1 fragment 6 H1486A	The mutation H1486A was introduced by two-step overlap PCR using oligonucleotides TATACTCGAGCAATTCGTCGCCTACCGCGAGC, TATATAGCTAGCGAACCGTGCCCCCCATTGCTCG, CAATACCCAGAATGCCTCGATGATGG, and CCATCATCGAGGCATTCTGGGTATTG and pB380 as the template. The PCR product was digested with XhoI and NheI, and ligated into 2473 YEpGal555 vector.	This study
pB631 YEpGal555 TcART	TccC3 with a 100 amino-acid long N-terminal deletion in 2473 YEpGal555 vector.	[14]
pB798 YEpGal555 Mcf1 1-881	Fragment of the *mcf1* gene, encoding amino acids 1-881, was PCR amplified using oligonucleotides TATAGGATCCATGGAACAGAAGTTGATTTCCGAAGAAGACCTCGCTTCTATATCCAAAGATTTTACG and ATATCTCGAGGCCCTGATTGATGATCGGCAAG from pB394, digested with BamHI and XhoI and ligated in the digested 2473 YEpGal555 vector.	This study
pB799 YEpGal555 Mcf1 1-431	Fragment of the *mcf1* gene, encoding amino acids 1-431, was PCR amplified using oligonucleotides TATAGGATCCATGGAACAGAAGTTGATTTCCGAAGAAGACCTCGCTTCTATATCCAAAGATTTTACG and ATATCTCGAGACGACCGGCAAAACCTAC from pB394, digested with BamHI and XhoI and ligated in the digested 2473 YEpGal555 vector.	This study
pB800 YEpGal555 Mcf1 432-881	Fragment of the *mcf1* gene, encoding amino acids 432-881, was PCR amplified using oligonucleotides tatagGATCCatggaacagaagttgatttccgaagaagacctcCAGTACTTGCTGGAAATGCC and ATATCTCGAGGCCCTGATTGATGATCGGCAAG from pB394, digested with BamHI and XhoI and ligated in the digested 2473 YEpGal555 vector.	This study
pB801 YEpGal555 Mcf1 216-431	Fragment of the *mcf1* gene, encoding amino acids 216-431, was PCR amplified using oligonucleotides tatagGATCCatggaacagaagttgatttccgaagaagacctcAAGGTGCCTCAGGCCTCCG and ATATCTCGAGACGACCGGCAAAACCTAC from pB394, digested with BamHI and XhoI and ligated in the digested 2473 YEpGal555 vector.	This study
pB802 YEpGal555 Mcf1 1-215	Fragment of the *mcf1* gene, encoding amino acids 1-215, was PCR amplified using oligonucleotides	This study

	TATAGGATCCATGGAACAGAAGTTGATTTCCGAAGAAGACCTCGCTTCTATATCCAAAGATTTTACG and ATATCTCGAGGGTCAGCGCACCGAGAGC from pB394, digested with BamHI and XhoI and ligated in the digested 2473 YEpGal555 vector.	
pB827 YEpGal555 Mcf1 1-980	Fragment of the *mcf1* gene, encoding amino acids 1-980, was PCR amplified using oligonucleotides TATAGGATCCATGGAACAGAAGTTGATTTCCGAAGAAGACCTCGCTTCTATATCCAAAGATTTTACG and tatactcgaGCTCAGTGTACTCAAGCTCATACAAG from pB394, digested with BamHI and XhoI and ligated in the digested 2473 YEpGal555 vector.	This study
pB828 YEpGal555 Mcf1 1-1080	Fragment of the *mcf1* gene, encoding amino acids 1-1080, was PCR amplified using oligonucleotides TATAGGATCCATGGAACAGAAGTTGATTTCCGAAGAAGACCTCGCTTCTATATCCAAAGATTTTACG and tatactcgaGCGCAGCGAGGCCCTCCGCGG from pB394, digested with BamHI and XhoI and ligated in the digested 2473 YEpGal555 vector.	This study
pB829 YEpGal555 Mcf1 1-1180	Fragment of the *mcf1* gene, encoding amino acids 1-1180, was PCR amplified using oligonucleotides TATAGGATCCATGGAACAGAAGTTGATTTCCGAAGAAGACCTCGCTTCTATATCCAAAGATTTTACG and tatactcgaGGGCCCACGCAGCAATCGCCGG from pB394, digested with BamHI and XhoI and ligated in the digested 2473 YEpGal555 vector.	This study
pB830 YEpGal555 Mcf1 1-1280	Fragment of the *mcf1* gene, encoding amino acids 1-1280, was PCR amplified using oligonucleotides TATAGGATCCATGGAACAGAAGTTGATTTCCGAAGAAGACCTCGCTTCTATATCCAAAGATTTTACG and tatactcgaGATCGGTGCGCTGCTTACCCC from pB394, digested with BamHI and XhoI and ligated in the digested 2473 YEpGal555 vector.	This study
pB831 YEpGal555 Mcf1 1-1380	Fragment of the *mcf1* gene, encoding amino acids 1-1380, was PCR amplified using oligonucleotides TATAGGATCCATGGAACAGAAGTTGATTTCCGAAGAAGACCTCGCTTCTATATCCAAAGATTTTACG and tatactcgaGTACCAAGCGGTCAAAGACACTGCC from pB394, digested with BamHI and XhoI and ligated in the digested 2473 YEpGal555 vector.	This study
pB832 YEpGal555 Mcf1 1-1480	Fragment of the *mcf1* gene, encoding amino acids 1-1480, was PCR amplified using oligonucleotides TATAGGATCCATGGAACAGAAGTTGATTTCCGAAGAAGACCTCGCTTCTATATCCAAAGATTTTACG and tatactcgaGCGCGAACATCGAGGTTCCGG from pB394, digested with BamHI and XhoI and ligated in the digested 2473 YEpGal555 vector.	This study
pB833 YEpGal555 Mcf1 1-1580	Fragment of the *mcf1* gene, encoding amino acids 1-1580, was PCR amplified using oligonucleotides TATAGGATCCATGGAACAGAAGTTGATTTCCGAAGAAGACCTCGCTTCTATATCCAAAGATTTTACG and tatactcgaGGACGCTGGACAGTTCCTCAAATC from pB394, digested with BamHI and XhoI and ligated in the digested 2473 YEpGal555 vector.	This study
pB834 YEpGal555 Mcf1 1-1630	Fragment of the *mcf1* gene, encoding amino acids 1-1630, was PCR amplified using oligonucleotides TATAGGATCCATGGAACAGAAGTTGATTTCCGAAGAAGACCTCGCTTCTATATCCAAAGATTTTACG and tatactcgaGGTGCTCTTGCGCCAGCCGGG from pB394, digested with BamHI and XhoI and ligated in the digested 2473 YEpGal555 vector.	This study
pB990 YEpGal555 *Burkholderia* lethal factor 1	Gene, encoding *Burkholderia pseudomallei* lethal factor 1 (Blf1, Uniprot Q63UP7), was codon-optimized for expression in *S. cerevisiae*, synthesized and cloned into 2473 YEpGal555 vector by XhoI and KpnI sites.	This study
pH009 YEpGal555 Mcf1 32-881	Fragment of the *mcf1* gene, encoding amino acids 32-881, was PCR amplified using oligonucleotides tatagGATCCatggaacagaagttgatttccgaagaagacctcATGAGTCCTGATGAGCGTAC	This study

	and ATATCTCGAGGCCCTGATTGATGATCGGCAAG from pB394, digested with BamHI and XhoI and ligated in the digested 2473 YEpGal555 vector.	
pH010 YEpGal555 Mcf1 66-881	Fragment of the *mcf1* gene, encoding amino acids 66-881, was PCR amplified using oligonucleotides tatagGATCCatggaacagaagttgatttccgaagaagacctcATGCAGGACAACCCC and ATATCTCGAGGCCCTGATTGATGATCGGCAAG from pB394, digested with BamHI and XhoI and ligated in the digested 2473 YEpGal555 vector.	This study
pH011 YEpGal555 Mcf1 92-881	Fragment of the *mcf1* gene, encoding amino acids 92-881, was PCR amplified using oligonucleotides tatagGATCCatggaacagaagttgatttccgaagaagacctcGGCACGCCGGC and ATATCTCGAGGCCCTGATTGATGATCGGCAAGfrom pB394, digested with BamHI and XhoI and ligated in the digested 2473 YEpGal555 vector.	This study
pH012 YEpGal555 Mcf1 140-881	Fragment of the *mcf1* gene, encoding amino acids 140-881, was PCR amplified using oligonucleotides tatagGATCCatggaacagaagttgatttccgaagaagacctcAATGCGACGACATTGAGAG and ATATCTCGAGGCCCTGATTGATGATCGGCAAG from pB394, digested with BamHI and XhoI and ligated in the digested 2473 YEpGal555 vector.	This study
pH013 YEpGal555 Mcf1 176-881	Fragment of the *mcf1* gene, encoding amino acids 176-881, was PCR amplified using oligonucleotides tatagGATCCatggaacagaagttgatttccgaagaagacctcAGCCAAAAATATGTGACGC and ATATCTCGAGGCCCTGATTGATGATCGGCAAG from pB394, digested with BamHI and XhoI and ligated in the digested 2473 YEpGal555 vector.	This study
pH014 YEpGal555 Mcf1 1-834	Fragment of the *mcf1* gene, encoding amino acids 1-834, was PCR amplified using oligonucleotides tatagGATCCatggaacagaagttgatttccgaagaagacctcGCTTCTATATCCAAAGATTTTACG and atatCTCGAGttaTTCATAACGCGCAGCG from pB394, digested with BamHI and XhoI and ligated in the digested 2473 YEpGal555 vector.	This study
pH016 YEpGal555 Mcf1 1-764	Fragment of the *mcf1* gene, encoding amino acids 1-764, was PCR amplified using oligonucleotides tatagGATCCatggaacagaagttgatttccgaagaagacctcGCTTCTATATCCAAAGATTTTACG and atatCTCGAGttaGTCATTCTTGAAGTCCGG from pB394, digested with BamHI and XhoI and ligated in the digested 2473 YEpGal555 vector.	This study
pH017 YEpGal555 Mcf1 1-735	Fragment of the *mcf1* gene, encoding amino acids 1-735, was PCR amplified using oligonucleotides tatagGATCCatggaacagaagttgatttccgaagaagacctcGCTTCTATATCCAAAGATTTTACG and atatCTCGAGttaCTCAGAGCTGGTATTGAG from pB394, digested with BamHI and XhoI and ligated in the digested 2473 YEpGal555 vector.	This study
pH018 YEpGal555 Mcf1 1-699	Fragment of the *mcf1* gene, encoding amino acids 1-699, was PCR amplified using oligonucleotides tatagGATCCatggaacagaagttgatttccgaagaagacctcGCTTCTATATCCAAAGATTTTACG and atatCTCGAGttaCTTGGAAAAGCTATCGGC from pB394, digested with BamHI and XhoI and ligated in the digested 2473 YEpGal555 vector.	This study
pH023 YEpGal555 Mcf1 913-1125	Fragment of the *mcf1* gene, encoding amino acids 913-1125, was PCR amplified using oligonucleotides tatagGATCCatggaacagaagttgatttccgaagaagacctcGCCGGGTTGACGTCTGTAGG and atatCTCGAGttaTTGACTGTCCGCCACCTGGATC from pB397, digested with BamHI and XhoI and ligated in the digested 2473 YEpGal555 vector.	This study

pH024 YEpGal555 Mcf1 913-1125 G1031C	The mutation G1031C was introduced by two-step overlap PCR using oligonucleotides CGCTATCGGGCTGCTCG, CGAGCAGCCCGATAGCG, tatagGATCCatggaacagaagttgatttccgaagaagacctcGCCGGGTTGACGTCTGTAGG and atatCTCGAGttaTTGACTGTCCGCCACCTGGATC and pB397 as the template. The PCR product was digested with BamHI and XhoI and ligated into digested 2473 YEpGal555	This study
**Plasmids for protein expression in *E. coli***		
pETDuet	*E. coli* vector for expression of two genes.	Novagen
1330 pET28 Iota-A	*Clostridium perfringens* Iota-A with N-terminal His-tag in pET28 vector	[12]
pB656 pET28 MBP-TcHVR ΔN100 (TcART)	TcHVR with the N-terminal deletion in fusion with MBP tag in pET28 vector	[14]
pB475 pET28 His-Arf3 Q71L	Gene, encoding human ADP-ribosylating factor 3 (Arf3; Uniprot P61204) with N-terminal deletion of 17 amino acids and mutation Q71L, was codon-optimized for expression *E. coli*, synthesized and cloned into pET28a vector by NdeI and BamHI sites.	This study
pB394 pET28 His-Mcf1	*Mcf1* gene (Uniprot Q8KT65) was PCR amplified from *Photorhabdus luminescens* strain akhurstii using oligonucleotides tatacatatgGCTTCTATATCCAAAGATTTTACGAAC and tatagagctcTTAGATGGCCCAAGGCAGCTCGA. The PCR product was digested with NdeI and SacI and inserted into digested pET28a vector.	This study
pB397 pET28 His-Myc-Mcf1-FLAG	N-terminal Myc and C-terminal FLAG tags were added to pB394 in three steps. First, we removed a stop codon at the end of *mcf1* gene in pB394 by PCR using oligonucleotides tatacatatgGCTTCTATATCCAAAGATTTTACGAAC and tatagagctcGATGGCCCAAGGCAGCTCGA and pB394 as the template. The PCR product was digested with NdeI and SacI and inserted into pET28a (resulting plasmid pB395).<br>Second, we annealed oligonucleotides encoding Myc-tag tagtgaacagaagttgatttccgaagaagacctctc and tagagaggtcttcttcggaaatcaacttctgttcac, and inserted them into NdeI-digested pB395 (resulting plasmid pB396)<br>Finally, we annealed oligonucleotides encoding FLAG-tag agattacaaggatgacgacgataagtaaagct and ttacttatcgtcgtcatccttgtaatctagct, and inserted into SacI-digested pB396.	This study
pB446 pET28 His-Myc-Mcf1_C1397A-FLAG	The mutation C1397A was introduced by two-step overlap PCR using oligonucleotides aactttaagaaggagatataccatgggcagcagc, CCGCAAGCTtgtcgacggagctttacttatcgtcgtc, GTGACCGATCTGGTGGCCGTGCTTATCCTTTGGTCAGGGCC, and CTGACCAAAGGATAAGCACGGCCACCAGATCGGTCACCGAC and pB397 as the template. The PCR product was digested with NcoI and HindIII, and ligated into digested pET28 vector.	This study
pB852 pETDuet Arf3 Q71L	Arf3 Q71L was PCR-amplified from pB475 with oligonucleotides TATACAtatgtcatggtctcatccacaattcgaaaagtcacgcattctgatggtgggcctggatgcgg and GCTAGTTATTGCTCAGCGG. PCR product was digested with NdeI and SalI, and ligated into NdeI/XhoI-digested pETDuet vector.	This study

pB858 pETDuet Arf3 Q71L His-Myc-Mcf1-FLAG	*Mcf1* gene was cut by NcoI and HindIII from pB397 and inserted into digested pB852.	This study
pB891 pET28 His-Mcf1-Myc	C-terminal Myc-tag was added to Mcf1 by annealing oligonucleotides cggatcaggtgaacagaagttgatttccgaagaagacctcta and agcttagaggtcttcttcggaaatcaacttctgttcacctgatccgagct and inserting them into SacI/HindIII-digested pB395	This study
pB914 His-Mcf1 Δ15C	C-terminal deletion was performed by PCR using oligonucleotides tatacatatgGCTTCTATATCCAAAGATTTTACGAAC and tatagagctcTTACCTGGTAAAACCCTTGACCAAGTG, and pB394 as the template. The PCR product was digested with NdeI and SacI and inserted into digested pET28a vector.	This study
pB915 His-Mcf1 C1397A Δ15C	The mutation C1397A was introduced by two-step overlap PCR using oligonucleotides tatacatatgGCTTCTATATCCAAAGATTTTACGAAC, tatagagctcTTACCTGGTAAAACCCTTGACCAAGTG, GTGACCGATCTGGTGGCCGTGCTTATCCTTTGGTCAGGGCC, and CTGACCAAAGGATAAGCACGGCCACCAGATCGGTCACCGAC and pB914 as the template. The PCR product was digested with NdeI and SacI and inserted into digested pET28a vector.	This study
pB916 pETDuet His-Myc-Mcf1-FLAG	*Mcf1* gene was cut by NcoI and HindIII from pB397 and inserted into the digested pETDuet vector.	This study
pB917 pETDuet Arf3 Q71L His-Myc-Mcf1_C1397A-FLAG	*Mcf1* gene was cut by NcoI and HindIII from pB446 and inserted into digested pB852.	This study
pB973 pET28 His-Mcf1 Y1286S Δ15C	The Y1286S mutation in the *mcf1* gene was introduced by two-step overlap PCR using oligonucleotides tatacatatgGCTTCTATATCCAAAGATTTTACGAAC, tatagagctcTTACCTGGTAAAACCCTTGACCAAGTG, GCGTGGGTGAACGTagCGCTATCTCTGAAG and CTTCAGAGATAGCGctACGTTCACCCACGC and pB914 as the template. The PCR product was digested with NdeI and SacI and inserted into digested pET28a vector.	This study
pB987 pET28 His-Mcf1 R957A Δ15C	The R957A mutation in the *mcf1* gene was introduced by two-step overlap PCR using oligonucleotides tatacatatgGCTTCTATATCCAAAGATTTTACGAAC, tatagagctcTTACCTGGTAAAACCCTTGACCAAGTG, CTGGTCAGATCGGGGgcCCTCCCGGCAGAGGG and CCCTCTGCCGGGAGGgcCCCCGATCTGACCAG and pB914 as the template. The PCR product was digested with NdeI and SacI and inserted into digested pET28a vector.	This study
pB989 pET28 His-Mcf1 Y1286S C1397A Δ15C	The C1397A mutation in the Mcf1 Y1286S variant was introduced by two-step overlap PCR using oligonucleotides tatacatatgGCTTCTATATCCAAAGATTTTACGAAC, tatagagctcTTACCTGGTAAAACCCTTGACCAAGTG, GTGACCGATCTGGTGGCCGTGCTTATCCTTTGGTCAGGGCC and CTGACCAAAGGATAAGCACGGCCACCAGATCGGTCACCGAC and pB973 as the template. The PCR product was digested with NdeI and SacI and inserted into digested pET28a vector.	This study
pB992 pET28 His-Mcf1 L1359G Δ15C	The L1359G mutation in the *mcf1* gene was introduced by two-step overlap PCR using oligonucleotides tatacatatgGCTTCTATATCCAAAGATTTTACGAAC, tatagagctcTTACCTGGTAAAACCCTTGACCAAGTG, CTGAGTACATCGATAAGGTAggGCGGCAGACTGCGG and	This study

	CCGCAGTCTGCCGCccTACCTTATCGATGTACTCAG and pB914 as the template. The PCR product was digested with NdeI and SacI and inserted into digested pET28a vector.	
pB993 pET28 His-Mcf1 L1359G R1360G Δ15C	The L1359G and R1360G mutations in the *mcf1* gene were introduced by two-step overlap PCR using oligonucleotides tatacatatgGCTTCTATATCCAAAGATTTTACGAAC, tatagagctcTTACCTGGTAAAACCCTTGACCAAGTG, CTGAGTACATCGATAAGGTAggGgGGCAGACTGCGGTATTC and GAATACCGCAGTCTGCCcCccTACCTTATCGATGTACTCAG and pB914 as the template. The PCR product was digested with NdeI and SacI and inserted into digested pET28a vector.	This study
pB996 pET28 His-Mcf1 L911A K912A Δ15C	The L911A K912A mutations in the *mcf1* gene were introduced by two-step overlap PCR using oligonucleotides tatacatatgGCTTCTATATCCAAAGATTTTACGAAC, tatagagctcTTACCTGGTAAAACCCTTGACCAAGTG, GGCGGTGAAGGATCTCGAAgccgcgGCCGGGTTGACGTCTGTAGG and CCTACAGACGTCAACCCGGCcgcggcTTCGAGATCCTTCACCGCC and pB914 as the template. The PCR product was digested with NdeI and SacI and inserted into digested pET28a vector.	This study
pB1000 pETDuet His-Myc-Mcf1-FLAG L911A K912A	The mutations L911A K912A were introduced by two-step overlap PCR using oligonucleotides aactttaagaaggagatataccatgggcagcagc, CCGCAAGCTtgtcgacggagctttacttatcgtcgtc, GGCGGTGAAGGATCTCGAAgccgcgGCCGGGTTGACGTCTGTAGG, and CCTACAGACGTCAACCCGGCcgcggcTTCGAGATCCTTCACCGCC and pB397 as the template. The PCR product was digested with NcoI and HindIII, and ligated into digested pETDuet vector.	This study
pB1004 pET28 His-Myc-Mcf1-FLAG L911A K912A I1271A Q1272A G1273A	The mutations I1271A Q1272A G1273A were introduced by two-step overlap PCR using oligonucleotides aactttaagaaggagatataccatgggcagcagc, CCGCAAGCTtgtcgacggagctttacttatcgtcgtc, CGGATTGACGATACAGCCgccgcagcCGGGGGTAAGCAGCGC and GCGCTGCTTACCCCCGgctgcggcGGCTGTATCGTCAATCCG and pB1000 as the template. The PCR product was digested with NcoI and HindIII, and ligated into digested pET28 vector.	This study
pB1006 pETDuet Arf3 Q71L His-Myc-Mcf1-FLAG L911A K912A	*Mcf1* gene with required mutations was cut by NcoI and HindIII from pB1000 and inserted into the digested pB852 vector.	This study
pB1008 pETDuet Arf3 Q71L His-Myc-Mcf1-FLAG L911A K912A I1271A Q1272A G1273A	*Mcf1* gene with required mutations was cut by NcoI and HindIII from pB1004 and inserted into the digested pB852 vector.	This study
pB1012 pET28 His-Myc-Mcf1-FLAG I1271A Q1272A G1273A	The mutations I1271A Q1272A G1273A were introduced by two-step overlap PCR using oligonucleotides aactttaagaaggagatataccatgggcagcagc, CCGCAAGCTtgtcgacggagctttacttatcgtcgtc, CGGATTGACGATACAGCCgccgcagcCGGGGGTAAGCAGCGC and GCGCTGCTTACCCCCGgctgcggcGGCTGTATCGTCAATCCG and pB397 as the template. The PCR product was digested with NcoI and HindIII, and ligated into digested pET28 vector.	This study

pB1022 pET28 His-Mcf1 ΔK907 Δ15C	The ΔK907 deletion in the *mcf1* gene was introduced by two-step overlap PCR using oligonucleotides tatacatatgGCTTCTATATCCAAAGATTTTACGAAC, tatagagctcTTACCTGGTAAAACCCTTGACCAAGTG, GCTGTTGGGGCAGGCGGTGGATCTCGAACTCAAG and CTTGAGTTCGAGATCCACCGCCTGCCCCAACAGC and pB914 as the template. The PCR product was digested with NdeI and SacI and inserted into digested pET28a vector.	This study
pB1023 pET28 His-Mcf1 ΔK907 ΔD908 Δ15C	The ΔK907 and ΔD908 deletions in the *mcf1* gene were introduced by two-step overlap PCR using oligonucleotides tatacatatgGCTTCTATATCCAAAGATTTTACGAAC, tatagagctcTTACCTGGTAAAACCCTTGACCAAGTG, CGCTGTTGGGGCAGGCGGTGCTCGAACTCAAGGCCG and CGGCCTTGAGTTCGAGCACCGCCTGCCCCAACAGCG and pB914 as the template. The PCR product was digested with NdeI and SacI and inserted into digested pET28a vector.	This study

Supplementary references

1. Aminzadeh, A., Larsen, C. E., Boesen, T. & Jørgensen, R. High-resolution structure of native toxin A from Clostridioides difficile . *EMBO Rep* **23**, (2022).
2. Luo, J. *et al.* TFPI is a colonic crypt receptor for TcdB from hypervirulent clade 2 C. difficile. *Cell* **185**, (2022).
3. Bullen, N. P. *et al.* An ADP-ribosyltransferase toxin kills bacterial cells by modifying structured non-coding RNAs. *Mol Cell* **82**, 3484-3498.e11 (2022).
4. Cruz-Migoni, A. *et al.* A Burkholderia pseudomallei toxin inhibits helicase activity of translation factor eIF4A. *Science* **334**, 821–824 (2011).
5. Lee, Y. *et al.* Makes caterpillars floppy-like effector-containing MARTX toxins require host ADP-ribosylation factor (ARF) proteins for systemic pathogenicity. *Proc Natl Acad Sci U S A* **116**, (2019).
6. Motlova, L., Klimova, N., Fiser, R., Sebo, P. & Bumba, L. Continuous Assembly of β-Roll Structures Is Implicated in the Type I-Dependent Secretion of Large Repeat-in-Toxins (RTX) Proteins. *J Mol Biol* **432**, (2020).
7. Harmat, V. *et al.* Structure and catalysis of acylaminoacyl peptidase: Closed and open subunits of a dimer oligopeptidase. *Journal of Biological Chemistry* **286**, (2011).
8. Dauden, M. I. *et al.* Molecular basis of tRNA recognition by the Elongator complex. *Sci Adv* **5**, (2019).
9. Schrödinger LLC. *The PyMOL Molecular Graphics System, Version~1.8*. (2015).
10. Krogh, A., Larsson, B., Von Heijne, G. & Sonnhammer, E. L. L. Predicting transmembrane protein topology with a hidden Markov model: Application to complete genomes. *J Mol Biol* **305**, (2001).
11. Heitman, J., Movva, N. R., Hiestand, P. C. & Hall, M. N. FK 506-binding protein proline rotamase is a target for the immunosuppressive agent FK 506 in Saccharomyces cerevisiae. *Proc Natl Acad Sci U S A* **88**, (1991).
12. Belyy, A. *et al.* Roles of Asp179 and Glu270 in ADP-ribosylation of actin by Clostridium perfringens Iota Toxin. *PLoS One* **10**, (2015).
13. Belyy, A. *et al.* The extreme C terminus of the Pseudomonas aeruginosa effector ExoY is crucial for binding to its eukaryotic activator, F-actin. *Journal of Biological Chemistry* **293**, (2018).
14. Belyy, A. *et al.* Mechanism of threonine ADP-ribosylation of F-actin by a Tc toxin. *Nat Commun* **13**, (2022).
