# Supplementary material for: Structure and activation mechanism of the Makes caterpillars floppy 1 toxin
Source: Nat Commun. 2023 Dec 12;14:8226. doi: 10.1038/s41467-023-44069-2 (PMC10716152; doi:10.1038/s41467-023-44069-2)
Supplement: Supplementary file 2 — Reporting Summary [file 41467_2023_44069_MOESM2_ESM.pdf]

nature portfolio

Corresponding author(s): Stefan Raunser

Last updated by author(s): Nov 18, 2023

Reporting Summary

Nature Portfolio wishes to improve the reproducibility of the work that we publish. This form provides structure for consistency and transparency in reporting. For further information on Nature Portfolio policies, see our Editorial Policies and the Editorial Policy Checklist.

Statistics

For all statistical analyses, confirm that the following items are present in the figure legend, table legend, main text, or Methods section.

n/a	Confirmed	
☐	☒	The exact sample size (n) for each experimental group/condition, given as a discrete number and unit of measurement
☐	☒	A statement on whether measurements were taken from distinct samples or whether the same sample was measured repeatedly
☒	☐	The statistical test(s) used AND whether they are one- or two-sided<br>*Only common tests should be described solely by name; describe more complex techniques in the Methods section.*
☒	☐	A description of all covariates tested
☒	☐	A description of any assumptions or corrections, such as tests of normality and adjustment for multiple comparisons
☒	☐	A full description of the statistical parameters including central tendency (e.g. means) or other basic estimates (e.g. regression coefficient) AND variation (e.g. standard deviation) or associated estimates of uncertainty (e.g. confidence intervals)
☒	☐	For null hypothesis testing, the test statistic (e.g. F, t, r) with confidence intervals, effect sizes, degrees of freedom and P value noted<br>*Give P values as exact values whenever suitable.*
☒	☐	For Bayesian analysis, information on the choice of priors and Markov chain Monte Carlo settings
☒	☐	For hierarchical and complex designs, identification of the appropriate level for tests and full reporting of outcomes
☒	☐	Estimates of effect sizes (e.g. Cohen's d, Pearson's r), indicating how they were calculated

Our web collection on statistics for biologists contains articles on many of the points above.

Software and code

Policy information about availability of computer code

Data collection	- Cryo-EM data: EPU version 2.7 and 2.8 (Thermo Fisher Scientific)<br>- SDS-PAGE and western blots: ImageLab version 5.2.1<br>- ITC: MicroCal PEAQ-ITC Control Software version 1.41
Data analysis	- Cryo-EM data: crYOLO version 1.6, 1.7, 1.8; CTFFIND version 4.1.13; MotionCor2 version 1.3; TranSPHIRE version 1.4, 1.5.13; SPHIRE version 1.4; Relion version 3.1; CryoSPARC version 4.1.0, PHENIX version 1.16, 1.17; ISOLDE version 1.4; UCSF Chimera version 1.14<br>- ITC: MicroCal PEAQ-ITC Analysis Software version 1.41<br>- rmsd calculation: PyMOL version 2.5.2

For manuscripts utilizing custom algorithms or software that are central to the research but not yet described in published literature, software must be made available to editors and reviewers. We strongly encourage code deposition in a community repository (e.g. GitHub). See the Nature Portfolio guidelines for submitting code & software for further information.

Data

Policy information about availability of data

All manuscripts must include a data availability statement. This statement should provide the following information, where applicable:

- Accession codes, unique identifiers, or web links for publicly available datasets
- A description of any restrictions on data availability
- For clinical datasets or third party data, please ensure that the statement adheres to our policy

The coordinates for the cryo-EM structures of the full-length Mcf1, Mcf1C1397A Δ15C, and the Mcf1C1397A Δ15C-Arf3 complex have been deposited in the Electron Microscopy Data Bank under accession numbers EMD-17440 [https://www.ebi.ac.uk/emdb/EMD-17440] (composite map; original and low-resolution consensus maps – EMD-17437 [https://www.ebi.ac.uk/emdb/EMD-17437], 17438 [https://www.ebi.ac.uk/emdb/EMD-17438], 17437 [https://www.ebi.ac.uk/emdb/EMD-17437], 17450 [https://www.ebi.ac.uk/emdb/EMD-17450]), EMD-17436 [https://www.ebi.ac.uk/emdb/EMD-17436] and EMD-17435 [https://www.ebi.ac.uk/emdb/EMD-17435]. The corresponding molecular models have been deposited at the wwPDB with accession codes PDB 8P52 [https://www.rcsb.org/structure/8P52], 8P51 [https://www.rcsb.org/structure/8P51] and 8P50 [https://www.rcsb.org/structure/8P50]. The raw data generated during the current study are available from the corresponding author in request. Source data are provided with this paper. Uncropped gels, western blots and corresponding loading controls can be found in Supplementary Fig. 11. We used the following previously published structures: 6II6 [https://www.rcsb.org/structure/6II6], 7POG [https://www.rcsb.org/structure/7POG], 7V1N [https://www.rcsb.org/structure/7V1N], 7RT7 [https://www.rcsb.org/structure/7RT7], 3TU8 [https://www.rcsb.org/structure/3TU8], 6SUS [https://www.rcsb.org/structure/6SUS], 3O4J [https://www.rcsb.org/structure/3O4J], 6QK7 [https://www.rcsb.org/structure/6QK7].

Research involving human participants, their data, or biological material

Policy information about studies with human participants or human data. See also policy information about sex, gender (identity/presentation), and sexual orientation and race, ethnicity and racism.

Reporting on sex and gender	N/A
Reporting on race, ethnicity, or other socially relevant groupings	N/A
Population characteristics	N/A
Recruitment	N/A
Ethics oversight	N/A

Note that full information on the approval of the study protocol must also be provided in the manuscript.

Field-specific reporting

Please select the one below that is the best fit for your research. If you are not sure, read the appropriate sections before making your selection.

☒ Life sciences ☐ Behavioural & social sciences ☐ Ecological, evolutionary & environmental sciences

For a reference copy of the document with all sections, see nature.com/documents/nr-reporting-summary-flat.pdf

Life sciences study design

All studies must disclose on these points even when the disclosure is negative.

Sample size	No sample size calculation was performed. The chosen sample sizes are sufficient and customary in the filed (Belyy A et al., Nat Commun 2022; Cherny KE et al., Anareobe 2022; Jurėnas D et al., Nucleic Acids Res 2022;)
Data exclusions	No data were excluded.
Replication	The experiments were replicated successfully and the information on the replicates are stated in the figure captions.
Randomization	No randomization was necessary as all data, which passed quality control, were used for analysis. Covariates were not controlled.
Blinding	This is not relevant to our study because we do not carry out statistic study.

Reporting for specific materials, systems and methods

We require information from authors about some types of materials, experimental systems and methods used in many studies. Here, indicate whether each material, system or method listed is relevant to your study. If you are not sure if a list item applies to your research, read the appropriate section before selecting a response.

Materials & experimental systems

n/a	Involved in the study
	☒ Antibodies
	☒ Eukaryotic cell lines
☒	☐ Palaeontology and archaeology
☒	☐ Animals and other organisms
☒	☐ Clinical data
☒	☐ Dual use research of concern
☒	☐ Plants

Methods

n/a	Involved in the study
☒	☐ ChIP-seq
☒	☐ Flow cytometry
☒	☐ MRI-based neuroimaging

Antibodies

Antibodies used

Myc-tag (9B11, reference #2276) mouse mAb Cell signaling technology, lot 24, reference 02/2019, dilution1:10000 or 1:2000.
RPS9 polyclonal rabbit antibody (used at dilution 1:10000), produced by Euogentec, is a present of Prof. S. Rospert (University of Freiburg).
Anti-pan-ADP-ribose binding reagent, reference #MABE1016 "a His-tagged recombinant protein fused to rabbit Fc tag", lot 3474103, dilution 1:3000.
Anti-Mcf1 polyclonal rabbit antibody (used at dilution 1:10000), custom-made by Cambridge research biochemicals.
Anti-FLAG clone M2 reference F3165 Sigma Aldrich lot SLBN5629C, dilution 1:5000.
Anti-His antibody reference H1029 Sigma Aldrich lot 025M4780V, dilution 1:3000.
Secondary anti-mouse HRP Bio-Rad, lot unknown, reference 1706516, dilution 1:3000.
Secondary anti-rabbit HRP Bio-Rad, lot unknown, reference 1706515, dilution 1:3000.

Validation

"Myc-tag (9B11) Mouse mAb detects recombinant proteins containing the Myc epitope tag. The antibody recognizes the Myc-tag fused to either the amino or carboxy terminus of targeted proteins in transfected cells. The antibody may cross-react with c-myc protein. The antibody may weakly cross-react with a protein of unknown origin ~90kDa." http://www.cellsignal.de/products/primary-antibodies/myc-tag-9b11-mouse-mab/2276

Anti-RPS9 antibody was validated by the side-by-side western blot analysis of the WT yeast strain and a strain with tagged RPS9 protein (Raue et al., JBC 2007; Zhang et al., Nat Commun 2021)

"Anti-pan-ADP-ribose binding reagent is useful for the affinity detection of both mono- and poly-ADP-ribosylated proteins on membranes in a manner similar to antibody-based Western and dot blot analysis" https://www.merckmillipore.com/DE/en/product/Anti-pan-ADP-ribose-binding-reagent,MM_NF-MABE1016

Anti-Mcf1 antibody was validated by the side-by-side western blot analysis WT bacteria and E. coli lysate expressing recombinant Mcf1.

"Anti-Flag M2 antibody is used for the detection of Flag fusion proteins. This monoclonal antibody is produced in mouse and recognizes the FLAG sequence at the N-terminus, Met N-terminus, and C-terminus. The antibody is also able to recognize FLAG at an internal site. M2, unlike M1 antibody is not Calcium dependent." https://www.sigmaaldrich.com/DE/en/product/sigma/f3165

"The Monoclonal Anti-polyHistidine (mouse IgG2a) antibody recognizes native or denatured, reduced forms of synthetic polyhistidine polyhistidine-tagged fusion proteins." http://ww.sigmaaldrich.com/DE/en/product/sigma/h1029

Eukaryotic cell lines

Policy information about cell lines and Sex and Gender in Research

Cell line source(s)	Sf9 insect cell line was purchased from Oxford Expression Technologies Ltd. Reference 600102.
Authentication	The Sf9 cell line was not authenticated.
Mycoplasma contamination	The cells were not tested for mycoplasma contamination.
Commonly misidentified lines (See ICLAC register)	No commonly misidentified cell lines were used.

Plants

Seed stocks	N/A
Novel plant genotypes	N/A
Authentication	N/A
