# Supplementary material for: Structure and activation mechanism of the Makes caterpillars floppy 1 toxin
Source: Nat Commun. 2023 Dec 12;14:8226. doi: 10.1038/s41467-023-44069-2 (PMC10716152; doi:10.1038/s41467-023-44069-2)
Supplement: Supplementary file 3 — Description of Additional Supplementary Files [file 41467_2023_44069_MOESM3_ESM.pdf]

Title: Supplementary Movie 1.
Description: Architecture of Mcf1.

Title: Supplementary Movie 2.
Description: Mechanism of activation of Mcf1.
